# Supplementary material for: Content-rich biological network constructed by mining PubMed abstracts
Source: BMC Bioinformatics. 2004 Oct 8;5:147. doi: 10.1186/1471-2105-5-147 (PMC528731; doi:10.1186/1471-2105-5-147)
Supplement: Additional File 2 — The original results of the above study (non-essential files are deleted to keep the file size under the limit set by BMC bioinformatics). [file 1471-2105-5-147-S2.bz2 › chilibotAdditionalFile2/dip10/142ID7739537E502/html/RBL1_TFDP1.html]

 


 **RBL1** and **TFDP1** 
  
Found 64 abstracts in PubMed, retrieved 10.  
 

 What does Google say? 
 PDF only 
| .edu only 

---

**Interactive relationship** (e.g. stimulation, inhibition, etc)

**Non-interactive relationship** (e.g. studied together, co-existance, homology, etc.)

- In serum stimulated S phase cells, the composition of the complex switched to E2F1 4, DP1  [ **TFDP1** ]  and p107  [ **RBL1** ] , together with cyclin A and cyclin dependent kinase 2.  Ref: 12139485 Biochem J, 2002
- Release of E2F1 DP1  [ **TFDP1** ]  heterodimers from repression mediated by the retinoblastoma tumor suppressor pRB  [ **RBL1** ]  triggers cell cycle entry into S phase, suggesting that E2F1 and DP1  [ **TFDP1** ]  proteins must act in unison, either to facilitate or to suppress cell cycle progression.  Ref: 12588846 Development, 2003
- Overexpression of c myc, Cyclins D1, E, and A, and E2F1 genes, at messenger RNA mRNA and protein levels, rise in Cyclin D1 CDK4, Cyclin E CDK2, and E2F1 DP1  [ **TFDP1** ]  complexes, and pRb  [ **RBL1** ]  hyperphosphorylation occurred in nodules and HCCs of F344 rats.  Ref: 12029619 Hepatology, 2002
- A complex containing Smad3, the transcription factors E2F4 5 and DP1  [ **TFDP1** ] , and the corepressor p107  [ **RBL1** ]  preexists in the cytoplasm.  Ref: 12150994 Cell, 2002
- Furthermore, E2F species were sequestered in complexes with p107  [ **RBL1** ] , p130, DP 1  [ **TFDP1** ] , and cyclins A and E.  Ref: 12234612 Biochem Pharmacol, 2002
- Moreover, introduction of constitutively active pRb  [ **RBL1** ]  and transcriptionally inert mutant E2F1 DP1  [ **TFDP1** ]  efficiently protected cardiomyocytes from apoptosis.  Ref: 12411392 Circ Res, 2002
